# Are fish fed with cyanobacteria safe, nutritious and delicious? A laboratory study

**DOI:** 10.1038/srep15166

**Published:** 2015-10-16

**Authors:** Hualei Liang, Wenshan Zhou, Yulei Zhang, Qin Qiao, Xuezhen Zhang

**Affiliations:** 1 Fisheries College of Huazhong Agricultural University, Freshwater Aquaculture Collaborative Innovation Center of Hubei Province, Wuhan 430070, People’s Republic of China

## Abstract

Toxic cyanobacterial blooms, which produce cyclic heptapeptide toxins known as microcystins, are worldwide environmental problems. On the other hand, the cyanobacteria protein (30–50%) has been recommended as substitute protein for aquaculture. The present laboratory study verified the feasibility of cyanobacteria protein substitution and risk assessment. Goldfish were fed diets supplemented lyophilised cyanobacteria powder for 16 weeks with the various doses: 0% (control), 10%, 20%, 30% and 40%. Low doses (10% and 20%) promoted growth whereas high doses (30% and 40%) inhibited growth. In cyanobacteria treated fish, the proximate composition of ash, crude fat content and crude protein content decreased in 16 weeks; the saturated fatty acid (SFA) content significantly increased; the n-3 polyunsaturated fatty acid content, collagen content and muscle pH significantly decreased; cooking loss percents increased significantly. Muscle fiber diameter and myofibril length were negatively correlation. Additionally, flavour compounds (e.g., amino acids, nucleotides, organic acids and carnosine) changed significantly in the treated fish, and odour compounds geosmin and 2-methylisoborneol increased significantly. The estimated daily intake (EDI) of microcystins in muscle was close to or exceeded the World Health Organization (WHO) tolerable daily intake (TDI), representing a great health risk. Cyanobacterie is not feasible for protein sources use in aquaculture.

Anthropogenic activities lead to the eutrophication in lakes, lagoons and reservoirs, and eutrophication has become a serious environmental threat worldwide. Eutrophic freshwater ecosystems increase the likelihood of cyanobacteria blooms. Toxic cyanobacterial blooms present great risks to animal and human health due to toxins released in water or accumulated in aquatic food, and they have become an important environmental problem^1^.

Of all the cyanobacterial toxins, microcystins (MCs), a family of cyclic peptide toxins, are the most toxic group and are also the most commonly encountered in a contaminated aquatic system. Nearly 80 different congeners have been identified so far^2^. MCs cause the inhibition of protein phosphatases 1 (PP1) and protein phosphatases 2A (PP2A)^3^. MCs have been widely reported to exert significant toxic effects in both animals and humans^4^. They are referred to as hepatotoxins because the liver is the primary target in cases of animal and human poisonings^5^. In 1996, the death of over 50 patients occurred in Caruaru, Brazil due to MCs contamination in haemodialysis water^6^. Due to its high toxicity, the maximum allowable concentration for MCs in drinking water was established as 1 μg l^−1^ day^−1^. The tolerable daily intake (TDI) value of 0.04 μg kg^−1^ of body weight day^−1^ was proposed as a provisional guideline value by the WHO^7^.

Cyanobacteria have been reported to contain 30–50% crude protein, polysaccharides and pigment nutrients. Due to the scarcity and cost of fish meal in the world market, it is imperative to reduce feed costs by exploring cheaper alternative protein sources for aquaculture feeds. Cyanobacteria have the potential to fill this need in fish feeds^8^, however the cyanobacterial toxin is a big problem.

In aquatic systems, where fish stand at the top of the food chain, it is well known that MCs can bioaccumulate in their bodies. MCs are stable and resistant to degradation even during cooking^9^. Therefore, the toxins can be transferred along the food web to higher trophic levels, even to humans. This is of great concern for public health because the chronic ingestion of MCs in food and drinking water has considerable potential to promote cancer^10^ and other risks to human health. Goldfish (*Carassius auratus*) is a dominant freshwater species and is widely consumed in China. MCs concentration in muscle was highest in omnivorous fish, followed by phytoplanktivorous fish, and was lowest in carnivorous fish^11^. Because the goldfish is an omnivorous fish, MCs can comprise a significant portion of its diet, which might lead to the accumulation of MCs. Therefore, it is important to evaluate the toxicity of MCs in fish as well as the toxic impact on human health caused by contaminated aquatic production.

The aims of the present study were to examine the chronic toxicity of the dietary intake of cyanobacteria in terms of the growth index, proximate composition, muscle quality, flavour compounds and odour compounds in goldfish and to find the relationship between dietary intake and accumulation of MCs in fish muscles. The present study will try to answer the following questions: with toxin-producing cyanobacteria as fish food, are the fleshes of fish safe, nutritious and delicious?

## Results

### Biometric data

Significant differences in wet weight, body length and total length were observed between treatment and control groups (Table 1). Wet weights of low doses diets (10% and 20%) were higher than those of control, but high doses diets (30% and 40%) were lower. Body length and total length in diets of 10% and 20% significantly increased, however, significantly decreased in 40% group. In this study, the fish fed with low doses diets promoted growth, but high doses diets inhibited it.

### Proximate composition

Table 1 showed that the ash contents of 30% group significantly decreased in 16 weeks, and 40% group significantly decreased in 12 and 16 weeks. Crude protein contents of 10% group were significantly decreased in 16 weeks, and 20% group significantly increased in 12 weeks but significantly decreased in 16 weeks. 30% group significantly increased in 4 and 8 weeks but significantly decreased in 16 weeks, and 40% group significantly increased in 8 weeks but significantly decreased in 16 weeks. Crude fat contents of 10% group significantly decreased in 16 weeks, 20% group significantly decreased in 12 and 16 weeks. 30% and 40% groups significantly decreased in 8, 12 and 16 weeks. No significant difference was found in moisture content among different groups.

Fatty acid composition of all the groups was shown in Table 2. The SFA levels of 10% group significantly increased in 16 weeks, 20% group significantly increased in 12 and 16 weeks, 30% group significantly increased in 8, 12 and 16 weeks, 40% group significantly increased in 4, 8, 12 and16 weeks. No significant alterations of the monounsaturated fatty acids (MUFA) levels were observed among all groups. The long chain n-3, n-6 fatty acids referred to polyunsaturated fatty acids (PUFA), the PUFA contents of 20% group significantly decreased in 12 weeks, 30% group significantly decreased in 16weeks, and 40% group significantly decreased in 12 and 16 weeks. The n-6 PUFA were no significant difference among all groups. The n-3 PUFA of 10% and 20% groups significantly decreased in 16 weeks, 30% and 40% groups significantly decreased in 8, 12 and 16 weeks. The percentages of C20:4 (Arachidonic acid, ArA), C20:5 (Eicosapentaenoic acid, EPA) and C22:6 (Docosahexaenoic acid, DHA) in treatment groups significantly lower than those of control group. The n-3: n-6 significantly decreased in 16 weeks in 30% and 40% groups.

### Meat quality index

Compared with the control group, changes in muscle fiber diameters, myofibrils length, cooking loss, total collagen and muscle pH were observed in all cyanobacteria treatment groups (Table 3). The muscle fiber diameters of 10% group significantly increased in 16 weeks, 20% and 30% groups significantly increased in 12 and 16 weeks, 40% group significantly increased in 8, 12 and 16 weeks. The myofibrils length of 10% group significantly decreased in 16 weeks, 20% and 30% groups significantly decreased in 12 and 16 weeks, and 40% group significantly decreased in 8, 12 and 16 weeks.

The cooking loss of 10% and 20% groups significantly increased in 16 weeks, 30% group significantly increased in 12 and 16 weeks, 40% group significantly increased in 8, 12 and 16 weeks. The total collagen of 10% and 20% groups significantly decreased in 16 weeks, 30% group significantly decreased in 12 and 16 weeks, and 40% group significantly decreased in 4, 8, 12 and 16 weeks. The muscle pH of 20% and 30% groups significantly decreased in 16 weeks, 40% group significantly decreased in 12 and 16 weeks.

### Flavor composition

The amino acid composition of all the groups was shown in Table 4. The total amino acid of 20%, 30% and 40% groups were significantly increased in 4 weeks, but 40% group significantly decreased in 16 weeks. The flavor amino acid of 20%, 30% and 40% groups significantly increased in 4 weeks, but 30% group significantly decreased in 12 and 16 weeks, 40% group significantly decreased in 8, 12 and 16 weeks.

The organic acids composition of all the groups was shown in Table 5. The oxalic acid of 20% group significantly increased in 8, 12 and 16 weeks, 30% and 40% groups significantly increased in 8 and 12 weeks. The lactic acid of 30% group significantly increased in 16 weeks, 40% group significantly increased in 12 and 16 weeks. The acetic acid of 10% and 20% groups significantly increased in 8, 12 and 16 weeks, 30% and 40% groups significantly increased in 4, 8, 12 and 16 weeks. The pyruvic acid of 20% group significantly increased in 8 weeks, 30% group significantly increased in 16 weeks, and 40% group significantly increased in 8 and 16 weeks. The propanoic acid of 20% group significantly increased in 12 and 16 weeks, 30% group significantly increased in 4 and 12 weeks, and 40% group significantly increased in 4, 8, 12 and 16 weeks. No significant difference was found in succinic acid content among different groups.

The nucleotide compounds composition of all the groups was shown in Table 6. The cytidine mono-phosphate (CMP) of 20% group significantly decreased in 16 weeks, 30% and 40% groups decreased in 12 and 16 weeks. The uridine mono-phosphate (UMP) of 30% group significantly decreased in 16 weeks, 40% group decreased in 12 and 16 weeks. The inosine monophosphate (IMP) of 10% and 20% groups significantly decreased in 16 weeks, 30% group significantly decreased in 12 and 16 weeks, and 40% group significantly decreased in 8, 12 and 16 weeks. The Guanosine mono-phosphate (GMP) of 20% group significantly decreased in 16 weeks, 30% and 40% groups decreased in 12 and 16 weeks. The adenosine 5′-triphosphate (ATP) of 10% group significantly decreased in 16 weeks, 20% group decreased in 12 and 16 weeks, 30% group decreased in 8, 12 and 16 weeks, 40% group decreased in 4, 8, 12 and 16 weeks. The adenosine 5′-diphosphate (ADP) of 10% and 20% groups significantly decreased in 16 weeks, 30% and 40% groups decreased in 12 and 16 weeks. The adenosine 5′-monophosphate (AMP) of 10% and 20% groups significantly decreased in 16 weeks, 30% group decreased in 12 and 16 weeks, and 40% group decreased in 8, 12 and 16 weeks. The hypoxanthine (Hx) of 10% and 20% groups significantly increased in 12 and 16 weeks, 30% and 40% groups increased in 8, 12 and 16 weeks. The inosine (HxR) of 20% group significantly increased in 16 weeks, 30% group increased in 12 and 16 weeks, 40% group increased in 12 and 16 weeks.

The carnosine composition of all the groups was shown in Table 7. The carnosine of 20% group significantly increased in 12 and 16 weeks, 30% and 40% groups significantly increased in 4, 8, 12 and 16 weeks.

### Odor composition

**Figure 1:**
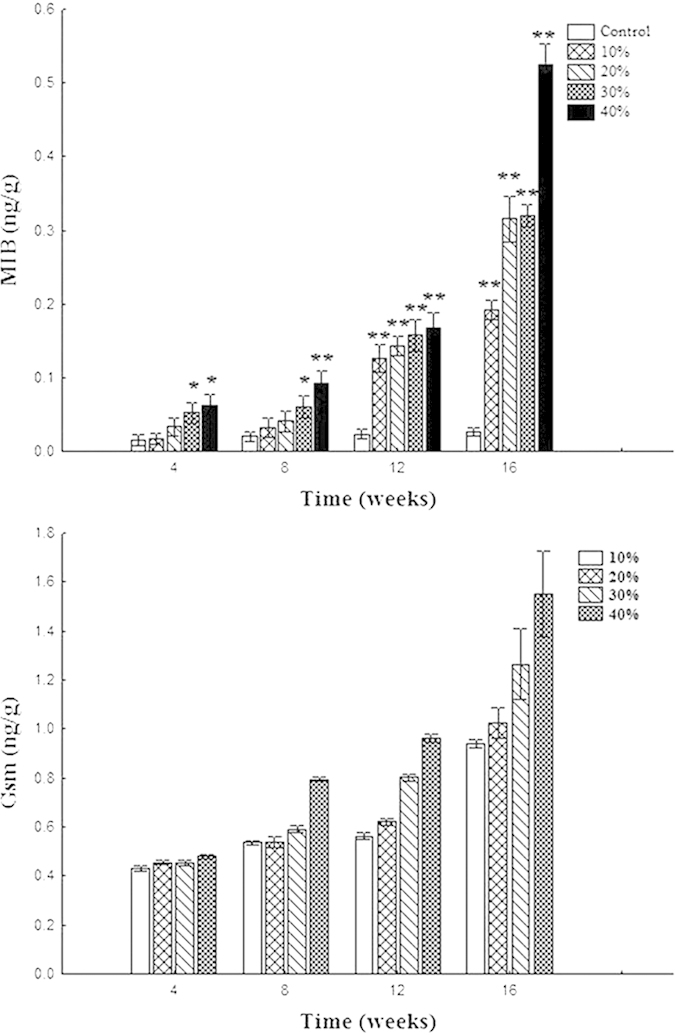
The MIB and GSM content of goldfish (*Carassius auratus*) after 4, 8, 12 and 16 weeks of feeding with diets containing cyanobacteria lyophilized powder. The GSM contents of control groups were not detected. The values were expressed as mean ± SD (n = 3). Differences were measured against control values and considered to be statistically significant at P < 0.05 level indicating with * and P < 0.01 level indicating **, respectively.

The MIB and GSM content of all groups in muscle was shown in Fig. 1. The GSM and MIB contents all increased with a dose-dependent effect.

### Histopathological observation

**Figure 2:**
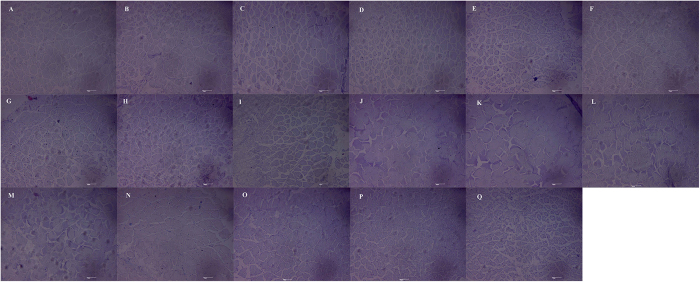
Light microscopy of muscle fibre transverse section from the back of goldfish (*Carassius auratus*) after 4, 8, 12 and 16 weeks of feeding with diets containing cyanobacteria lyophilized powder. Control (**A**); the muscle fibres in 10%–40% dose group at 4 weeks (**B**–**E**): enlarged fibre spacing and disorderly arrangement; the muscle fibres in 10%–40% dose group at 8 weeks (**F**–**I**): enlarged fibre spacing, disorderly arrangement and partial degenerative muscle fibres; the muscle fibres in 10%–40% dose group at 12 weeks (**J**–**M**): disarray of muscle fibres and destroy of sarcoplasmic reticulum; the muscle fibres in 10%–40% dose group at 16 weeks (**N–Q**): disarray of muscle fibres, degenerative muscle fibres and destroy of sarcoplasmic reticulum.

Light microscopy of muscle fiber transverse section was observed (Fig. 2). The architecture of control group was intact with regular myofiber arrangement and separated by sarcoplasmic reticulum (Fig. 2A). In cyanobacteria treatment groups, disorganizations of cell structure and loss of adherence between muscle fibers were pronounced (Fig. 2B–Q). At 4 and 8 weeks, the muscle fibers damage mainly included enlarged fiber spacing, disorderly arrangement and partial degenerative muscle fibers (Fig. 2B–I). At 12 weeks, disarray of muscle fibres and destroy of sarcoplasmic reticulum were the most prominent features (Fig. 2J–M). Histologically, disarray of muscle fibres, degenerative muscle fibres and destroy of sarcoplasmic reticulum were obvious in the 16 weeks (Fig. 2N–Q).

### MCs concentration in muscle and risk assessment

**Figure 3:**
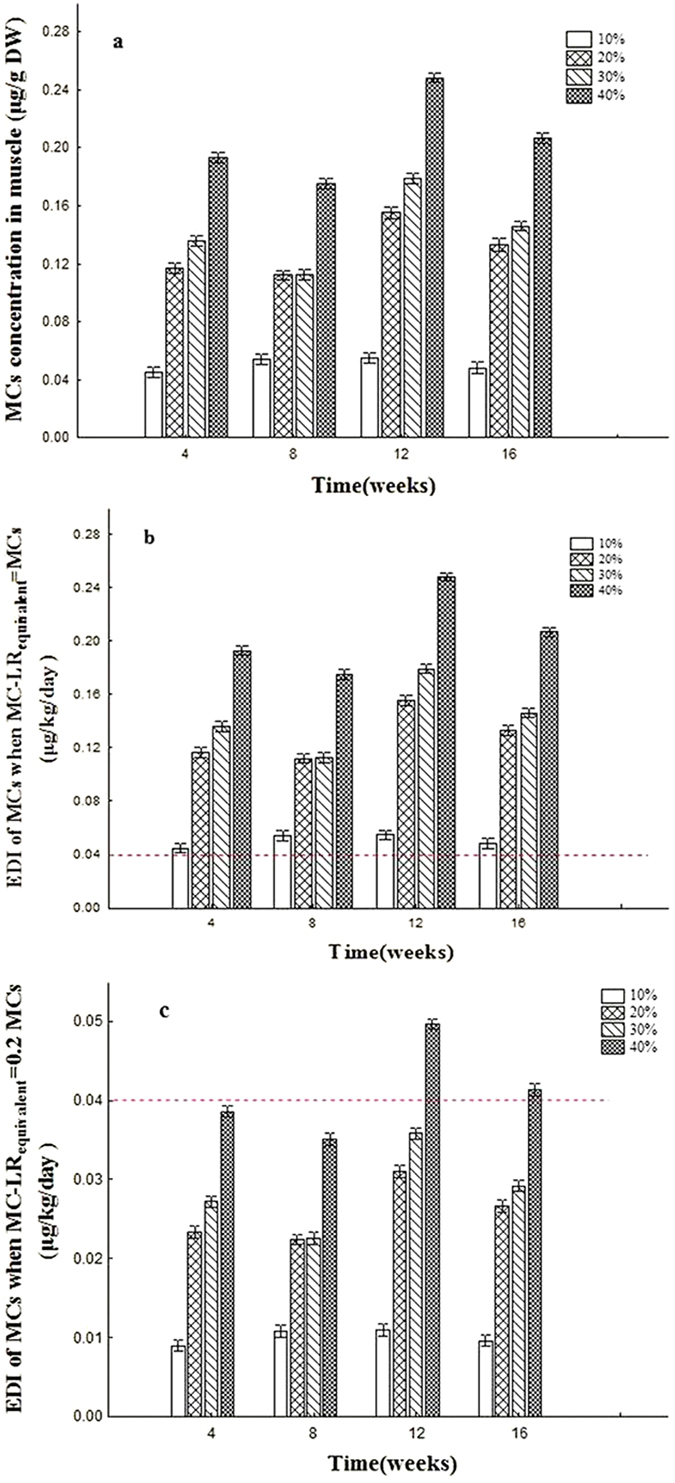
(**a**) MCs concentration in muscle of goldfish *(Carassius auratus)* after 4, 8, 12 and 16 weeks of feeding with diets containing cyanobacteria lyophilized powder. (**b**) EDI of microcystins by a weighing 60 kg person consumed 300 g muscle of goldfish *(Carassius auratus)* in each experimental group, when MCs was inferred as all MC-LR. The horizontal line indicates the maximum tolerable daily intake for humans (0.04 μg kg^−1^ day^−1^) proposed by the WHO. (**c**) EDI of microcystins by a person weighing 60 kg consumed 300 g muscle of goldfish *(Carassius auratus)* in each experimental group, when MCs was inferred as all MC-RR. The horizontal line indicates the maximum tolerable daily intake for humans (0.04 μg kg^−1^ day^−1^) proposed by the WHO.

The MCs accumulation in muscle of goldfish during the experiment and the estimated daily intake (EDI) of each group were shown in Fig. 3. The highest MCs concentration in muscle was observed at 12 weeks in 40% group, when it was detected 0.25 μg g^−1^. MCs contents exhibited a dose-dependent effect in cyanobacteria treatment groups. EDI values were obtained for evaluating the human health risk according to TDI level of WHO.

## Discussion

Toxic cyanobacterial blooms has become a serious global environmental concern. Feeding or ingestion of these toxic organisms, microcystins could be accumulated in fish through food chain and become a threat to human food safety^12^. Cyanobacteria have been reported to contain 30–50% crude protein, and it has been suggested as an alternative protein in aquaculture feeds^8^. However, whether the fish flesh safety and quality have been influenced should be well elucidated. The current laboratory study was carried out to investigate the effect of dietary intake of cyanobacteria on toxin accumulation in fish and assess the possibility of cyanobacteria as protein resource.

### Biometric data

Our study indicated that the dietary intake of cyanobacteria has a hormesis effect on the growth of goldfish. Hormesis is a dose-response relationship phenomenon characterised by low-dose stimulation and high-dose inhibition^13^. This promotion of the growth was probably due to many of the proteins, polysaccharides and pigment nutrients of cyanobacteria, and similar results were observed in Nile tilapias which were treated with cyanobacteria^12^. However, the growth inhibition may have been due to the protease antagonists in cyanobacteria, which may be not completely digested their diets^14^. The growth inhibition could also have resulted from a series of effects triggered by the toxicity of MCs^15^,^16^.

### Proximate composition

The proximate composition of the goldfish included the crude protein, crude fat, moisture and ash. In the present study, significantly decreased crude protein, crude fat and ash were observed in cyanobacteria groups. Miroslava^17^ also revealed the decrease of total fat in Nile tilapia exposed to cyanobacterial bloom. These decreases of body components may be due to nutritional imbalance and toxin of cyanobacteria in feeds and result into synthesis obstruction of protein and fat in fish. Microcystins could lead to the protein degradation and produce extensive damages in lipid composition^18^,^19^.

The decrease of n-3 PUFAs in the treatment groups may reduce the nutritional quality of their lipid components^20^. Additionally, the significant increase in SFAs and the decrease in the n-3 PUFAs indicate higher oxidation in the treatment groups^21^. In the present study, ArA, EPA and DHA significantly decreased in the exposed fish, possibly due to the presence of hydrogen peroxide. The reduction of these fatty acid augment the oxidised lipid products in fish when they were exposed to excessive oxidants (natural or man-made), which compromises the quality of fish oil for human to consumption^22^. The n-3:n-6 fatty acid ratio has been suggested to be a useful indicator for comparing the relative nutritional values of fish oils^23^. Increasing the n-3:n-6 fatty acid ratio in the human diet is essential to help prevent coronary heart disease by reducing plasma lipids and to help reduce cancer risk^24^. In our results, the n-3:n-6 ratio in high doses significantly decreased at high cyanobacteria exposure levels, indicating that the fish may not be suitable for human consumption.

### Meat quality

In the present study, increased muscle fibre diameter and decreased myofibril lengths were observed in cyanobacteria treatment groups. It is possible that the myofiber size and density influenced the folding endurance of the muscle fibers. Growth characteristics are influenced by genetics, diet, feeding regime and environmental conditions, which, in turn, affect the number and size distribution of the muscle fibers present in the fillet^25^. The size and number of muscle fibers are factors that influence muscle mass and meat quality^26^. Thus, cyanotoxins in the diets may have increased the muscle fiber diameters but decreased the folding endurance of the muscle fibers, significantly affecting the meat quality of the fish.

We observed a significant increase in cooking losses and collagen content, and a significant decline in muscle pH in cyanobacteria treated fish. The increase in cooking losses was probably caused by denaturation of degraded cytoskeletal proteins induced by MCs. Cytoskeletal degradation would lead to structural breakdown of the muscle^27^. The lower collagen content can be attributed to their lower muscle fiber density^28^. In addition, Muscle pH is an important post-mortem meat quality that reflects technological quality and can also influence the sensory quality of fresh meat. Rapid post-mortem glycolysis causes an accumulation of lactate, and the lactate formed cannot be removed, causing a decline in muscle pH. This decline in pH strongly affects muscle protein denaturation and, subsequently, meat quality^29^.

### Flavor composition

The taste of meat can be separated into distinct components: saltiness, sweetness, sourness and umami. The major taste-active components in meat are salts that provide saltiness, sugars that provide sweetness, organic acids that provide sourness, and free amino acids, nucleic acids and peptides that provide umami^30^. The components related to umami, such as amino acids, IMP and peptides have been considered as important contributors to the sensory quality of meat^31^. Amino acids are quality indicators for various fish and crustacean species. Glutamic acid, aspartic acid, alanine and glycine are responsible for flavour and taste in fish^32^. In the present study, the flavour amino acids of the high-dose groups were poorer than that of the control groups.

The organic acids in fish and fishery products are detected as oxalic, lactic, acetic, malic, propanoic, pyruvic, and succinic acids and fumarate^33^. The concentrations of organic acids in food influence the balance of the flavour, chemical stability, pH and, thus the quality of the food^34^. The acetic acid concentration significantly increased in the treatment groups, possibly because of the metabolism of lactose by lactic acid bacteria, the metabolism of citric and lactic acid, the catabolism of amino acids, or the oxidation of aldehydes and alcohols^35^. The significantly increased lactic acid in our results may be due to glycogen breakdown (glycolysis). The significantly increased pyruvic acid in the treatment groups, may have occurred because pyruvic acid is produced by lactic acid bacteria as an intermediate of both glucose and citrate metabolism^35^. Succinic acid has been reported to be one of the main taste-active compounds in some seafood^36^, but there were no significantly changes observed in treatment groups.

Nucleotide compounds are related to sensory attributes^37^. In fish muscle after slaughter, ATP rapidly breaks down to ADP and AMP, with the subsequent accumulation of IMP. The IMP is hydrolysed by autolytic enzymes (5′-nucleotidase, N) to HxR, which, in turn, is degraded to Hx by autolytic enzymes and/or microbial action^38^. It is well documented that ATP, the main adenine nucleotide in live fish, undergoes a rapid degradation to IMP after death, but IMP conversion to HxR proceeds at a slower rate^39^. The accumulation of HxR and Hx has been suggested to be related to both autolytic and microbial action^40^. This may be the reason that HxR and Hx were found to be significantly increased, whereas ATP, ADP and AMP were significantly decreased in our results.

AMP imparts an umami taste to the muscles of squid, abalone, scallops and short-necked clams^41^ and CMP is considered to be a major taste component for *Glyptocidaris crenularis*^31^. IMP and GMP are intensely flavuor-enhancers for the umami taste, and they have a much stronger umami flavour-enhancing capacity than monosodium glutamate^42^. In our study, IMP, CMP and GMP significantly decreased, indicating that the cyanobacteria in the diet may affect the umami flavor of the fish meat. Hx, together with some amino acids and peptides, may contribute to a bitter taste in meat^43^. Hx accumulation is associated with the progressive loss of desirable fresh fish flavour. It produces a bitter off-taste; therefore, it has been reported that Hx can be used as an index of fish freshness and taste quality^44^. HxR and Hx significantly increased in our treated groups, possibly indicating that bitter taste increased in the treated fish meat.

Carnosine was discovered at the beginning of the century in skeletal muscle, and it is presents in the muscle tissues of most vertebrate species, with the exception of certain fish^45^. The carnosine molecule is a scavenger of free-radical intermediates that binds hydrogen peroxide. It has been shown that carnosine is antioxidant preventing lipid peroxidation in linoleic acid systems^46^. Carnosine also has been postulated to act as a buffer to neutralise lactic acid produced in skeletal muscle that is undergoing anaerobic glycolysis^47^. Carnosine significantly increased in our study, possibly due to the increased oxidative stress and lactic acid content.

### Odor composition

GSM and 2-MIB are known to be mainly produced by cyanobacteria, and these off-flavours are present in the waters, and accumulate in fish muscles^48^. Because of their low odour detection threshold, many drinking water testing laboratories require detection limits of 1–3 ng l^−1^ and quantitation at 5 ng l^−1^ 49. Studies have detected MIB/GSM in various species of fish but have concluded that they do not result in any toxicity to either the fish or to humans through consumption of the fish^50^. Although taste and odour are rarely associated with toxic contaminants, for consumers, they are the primary ways to judge the quality and safety of fish, and it undermines consumer trust in the fish meat quality if odour and taste are compromised. Therefore, the increased odour detected in our study would have a negative effect on consumption.

### Histopathological observation

Muscle fiber degeneration with myocytolysis, enlarged fiber spacing, and disorderly arrangements were observed, and similar histopathological changes have also occurred in rats exposed to MC-LR^51^. The size and number of muscle fibers are factors that influence muscle mass and meat quality, and the morphological and biochemical characteristics of muscle fibers are major factors that influence energy metabolism and contribute to the sensory qualities of cooked meat from different species^52^. Collagen is the major component in the connective tissue of muscle in fish. The lower muscle fiber density leads to a lower content of collagen, and muscle structure changes in the water-holding properties will change the juiciness of the meat. Our laboratory study shows that cyanobacteria consumption had a negative impact on the quality of the fish muscle.

### MCs concentration in muscle and risk assessment

The bioaccumulation of cyanotoxins in aquatic animals, including fish, has been reported^53^. It has been suggested that accumulated MCs in edible fish may represent a risk to human health and MCs contaminated fish muscle is unfit for human consumption^54^. TDI is defined as the acceptable amount of a potentially toxic substance that can be consumed daily^55^. In the present study, sum of microcystins (mainly MC-LR, -RR, and -YR) were determined using ELISA method, which has been preferentially recommended for MCs detection^56^. Different microcystin variants exert various toxicity on animals, and MC-RR and MC-YR toxicity is about 0.2 and 0.4 times of MC-LR^57^. In order to compare the sums of MCs in goldfish muscle with MC-LR limits of WHO, we would like to propose a hypothesis that if the MCs are all MC-LR, so MC-LR_equivalent_ = MCs; and if the MCs are all MC-RR, then MC-LR_equivalent_ = 0.2MCs. Therefore, we can get a content range of MC-LR_equivalent_ (0.2MCs ~ MCs), which would make it possible to compare the muscle MCs with MC-LR limits recommended by WHO. The ratio of LR/RR = 0.42 in muscle of big head carp in Lake Taihu has been determined with electrospray ionization liquid chromatograghpy mass spectrum system (ESI-LC/MS^2^)^54^. MCs content in muscle of *Carassius auratus* was all MC-LR (i.e. MCs = MC-LR)^58^. Therefore, we inferred that the EDI of MC-LR_equivalent_ would far above 0.2MCs, indicating that MC-LR_equivalent_ of more muscle samples would exceeded the TDI value. So after chronic cyanobacteria dietary treatments in goldfish, the content of MC-LR_equivalent_ of muscle has been close to or above the MC-LR limits recommended by WHO, especially in 40% group at 16 weeks, Similarly, Zhao *et al.*^12^, also reported that Nile tilapia fed on toxic cyanobacteria is not suitable for human food. In laboratory conditions, fish fed with toxic cyanobacteria tend to accumulate more MCs than in natural waters, mainly due to no food choice and restricted living area. Therefore, the commercialization of fish and other animals fed with manufactured feeds added with toxic cyanobacteria powder could represent a risk to human health.

In conclusion, the results of our study indicate that low doses of cyanobacteria in the fish diet promoted growth, but high doses inhibited growth. More important, the present findings well answered our initial questions: in laboratory conditions, fish fed with toxic cyanobacteria are not edible due to MCs accumulation, are of low nutritional values and are not delicious. Therefore, we can draw a conclusion that cyanobacteria powder collected from blooms cannot used as substitute protein resource in aquaculture.

## Methods

All experiments were approved by and carried out in accordance with the guidelines of the Institutional Animal Care and Use Committees (IACUC) of Huazhong Agricultural University, Wuhan, China.

### Experimental setup and acclimation of fish

All studies were conducted using female goldfish (*Carassius auratus*) with a mean initial weight of 56.3 ± 1.08 g, mean initial body length of 11.04 ± 0.06 cm, mean initial total length of 13.08 ± 0.08 cm. They were purchased from a fish hatchery in Wuhan City, China. The fish were kept in a 150 L aquarium (95 cm L × 55 cm W × 40 cm H) containing dechlorinated tap water. During the trial, dissolved oxygen, pH and water temperature were measured daily. The experiment was conducted at a water temperature of 25 ± 2 °C, a dissolved oxygen (DO) concentration between 6.0 ~ 7.1 mg l^−1^ by continuously aerating, and a 12-h light: 12-h dark photoperiod. Ten percent of the tank water volume was replaced by fresh and dechlorinated water daily. The fish were fed with commercial fish food at a rate of 1% of the body weight twice a day (feeding ratio was 2% of body weight per day) and were acclimatized for 2 weeks before the beginning of the experiments. Feeding was terminated 48 h before the initiation of the experiment.

### Collection of the microcystis in the cyanobacterial bloom and measurement of microcystins

The cyanobacteria (blue-green algae) were collected from the surface blooms of Dianchi Lake, Yunnan, China. With microscope observation, 90% of the cyanobacteria biomass were *M. aeruginosa*.

The fresh algae was freeze-dried and stored at −70 °C until used. Crude algal matter was extracted three times with 75% (v/v) methanol and suspended in distilled water for the toxicity experiment. The MC concentration was analysed via high-performance liquid chromatography (HPLC, LC-10A, Shimadzu Corporation, Nakagyo-ku, Kyoto, Japan) equipped with an ODS column (Cosmosil 5C18-AR, 4.6 × 150 mm, Nacalai, Japan) and a SPA-10A UV Vis spectrophotometer set at 238 nm^59^. To determine the MC concentrations, the peak areas of the test samples were compared with those of the standards available (MC-LR, MC-RR and MC-YR Wako Pure Chemical Industries, Japan). The MC content was 1.41 mg g^−1^ dry weight (DW), among which MC-RR, -LR and -YR were 0.84, 0.50 and 0.07 mg g^−1^ DW, respectively.

### Diet preparation and exposure

After acclimatisation for 2 weeks, fish were exposed orally to the diets. Four diets containing 10%, 20%, 30% and 40% of cyanobacteria lyophilized powder and the control group diet without cyanobacteria were prepared (Table 8). The protein content of cyanobacteria is close to soybean meal (oil-extracted), so soybean meal (oil-extracted) was used as the substitute for cyanobacteria in the control diet. All diets were made into small sticky pellets by extrusion machine, air-dried and stored at 4 °C. The pellets were put into the tank, and they fell down on the bottom of tank for the fish to eat. It was ensured that fish ate up all pellets within 1 h. The MCs intake in the 10%, 20%, 30% and 40% groups were 2.82, 5.64, 8.46 and 11.28 mg kg^−1^ per day, respectively.

300 fish were randomly selected, divided into five groups and treated for 16 weeks as follows: group 1 served as the control, group 2 to group 5 were fed with 10%, 20%, 30% and 40% cyanobacteria lyophilised powder in their diets. During the experiment, the fish were provided with adequate aeration (DO 6.0 ~ 7.1 mg l^−1^) and fed at a rate of 1% of body weight twice a day at 9:00 and 16:00 (feeding ratio was 2% of body weight per day) for 16 weeks. No fish died during the experiment.

In each group, 60 fish were maintained in triplicate set (that is 20 fish in one aquarium), and at each sampling point (4, 8, 12 and 16 weeks), 9 fish were selected completely randomly from each group which meant 3 fish were randomly sampled out of each tank at each sampling point (n = 3). Fish were anaesthetised by immersion in water containing 100 mg l^−1^ tricaine cethane sulphonate (MS-222, Fluka Chemical Industries, Germany). Boneless muscles, to be used for analysis, were collected from above the lateral line and below the dorsal fin of the fish; the fillets were skinned, and stored in aplastic bag and kept in a refrigerator at −80 °C.

### Biometric parameters

At each sampling point, 3 fish were randomly sampled from each tank for biometric parameters determination. Wet weight was determined on a fine balance. All weights were determined in grams. Body length, which was measured to the nearest mm using a centimetre ruler refers to the length of the fish measured from the tip of the snout to the posterior end of the last vertebra or to the posterior end of the midlateral portion of the hypural plate. Total length refers to the length of the fish measured from the tip of the snout to the tip of the longer lobe of the caudal fin, usually measured with the lobes compressed along the midline.

### Proximate composition analysis

The muscles from each group were frozen until being used for the measurement of proximate body composition parameters (moisture, ash, crude protein and fat). The sample contents were measured according to Association of official Analytical Chemists^60^.

The lipids of the muscle samples were extracted with hexane–isopropanol (3:2 v/v), according to the method of Hara and Radin^61^. Fatty acid methyl esters (FAME) were analysed on an Agilent 6890N model gas chromatograph (GC), equipped with a flame ionisation detector (FID) and fitted with a capillary column (60 m, 0.25 mm i.d. and 0.25 μm). The individual fatty acid peaks were identified by comparing the of retention times with those of known mixtures of standard fatty acids (FAME, Sigma Chemical Industries, USA) run under the same operating conditions. Fatty acid percentages were calculated by dividing the peak area of a particular acid by the total area of all fatty acid peaks identified on the chromatograms.

### Meat quality measurement

For measuring muscle fiber diameters small pieces of muscle (approximately 3 × 3 × 10 mm) were cut from the back of the fish and put into the 20% HNO_3_ solution to soak for 24 h. A portion of the muscle was then dissected out and placed onto a slide, where a drop of glycerine was added. The sample was then observed under the microscope (10 × 40), and the muscle fiber diameters were recorded. For each group, 50 fibers were analysed, and the average diameter was calculated.

The lengths of the myofibrils were measured by using 0.5 g of muscle from each group, to which 5 mL of solution (KCl 14.90 g, EDTA-2Na 3.44 g, Boric acid 4.78 g in 2 L distilled water, pH 7.0) was added. The sample was then homogenised at 3000 r min^−1^ in 3 min, and a drop of the homogenate was placed on a slide. The length of 50 myofibrils was measured under the microscope (10 × 40), and the average was calculated.

Cooking loss was expressed as a percentage of the initial sample weight, and measured according to the methodology described by Choe^62^.

Total collagen was measured according to the method described by Suárze^63^ and was based on measuring the hydroxyproline content in the muscle samples.

Muscle pH was measured in the white muscle tissue from the dorsal side of the fish. The samples were homogenised with 10 volumes of deionised water (w/v)^64^, and pH was measured with a pH-meter (CG842 Schott, Germany).

### Flavor composition analysis

The amino acid contents of the samples were measured according to the method of Ning^65^, and the pH-adjusted samples were analysed by an Auto-matic Amino Acid Analyzer (L-8800, Hitachi, Japan). All measurements were performed in triplicate.

Carnosine, organic acids and nucleotides were all analysed using an Agilent 1200 HPLC, the column used with a C18 columns (250 mm length × 4.6 mm I.D.).

The samples used for the carnosine analysis were prepared according to the method detailed by Cornet^66^. The supernatant was collected and filtered through a 0.22 μm Millipore membrane filter and stored at −20 °C until detection. Chromatography was performed at 30 °C with a mobile phase which was composed of phase A (water) and phase B (acetonitrile) at the ratio of 80:20 and a constant flow rate of 1.0 mL min^−1^. Carnosine was detected by monitoring the UV absorbance at 215 nm, and the standard was purchased from Sigma Chemical Industries, USA.

All muscle samples from each group were used for organic acid analysis. A 2 g muscle sample was homogenised on ice with 20 mL of mobile phase 0.5% (w/v) (NH_4_)_2_HPO_4,_ pH 2.8. The homogenates were centrifuged at 6000 g for 10 min at 4 °C. The supernatant was collected and filtered through a 0.45-μm Millipore membrane filter and stored at −20 °C until detection. The mobile phase consisted of 0.5% (w/v) (NH_4_)_2_HPO_4_ adjusted to pH 2.80 ± 0.02 with H_3_PO_4_ (Solvent A) and acetonitrile (Solvent B), and the flow rate was set at 1.0 mL min^−1^ at 25 °C. The eluted acids were detected by monitoring their UV absorbance at 210 nm^67^. All organic acid standards were purchased from Sigma Chemical Industries, USA, including oxalic, pyruvic, lactic, acetic, succinic and propionic acids. Individual organic acids were quantified on the basis of the external standard method.

For the nucleotides analysis 5 g of muscle from each group was homogenized with 15 mL of cold 0.6 M perchloric acid in an ice bath. The extract was centrifuged at 10,000 g for 20 min at 4 °C, and the supernatant was neutralised (pH 6.5–6.8) by adding 1 M KOH and letting it stand in an ice bath for 20 min. The neutralised extract was centrifuged again as above, and the supernatant was collected and filtered through a 0.45-μm Millipore membrane filter and stored at −20 °C until use. Before the HPLC analysis, the thawed extract was centrifuged again for 5 min^68^. The composition of the mobile phase was as follows: solvent A was composed of 0.04 M KH_2_PO4 + 0.006 M K_2_HPO4, pH 7; solvent B was acetonitrile and the flow rate was set at 1.0 mL min^−1^ at 30 °C. The eluent was monitored at 254 nm. All nucleotide standards were obtained from the Sigma Chemical Industries, USA, including ATP, ADP, AMP, IMP, HxR, Hx, CMP, UMP and GMP.

### Odour composition analysis

For the odour composition analysis, 3 g muscle sample was minced and put into a 20 mL headspace vial, where 7 mL NaCl saturated solution, an internal standard and 5 ng 1-chlorodecane were added, and the vial was sealed with a screw cap. CAR/PDMS headspace solid phase microextraction (SPME) fibres were inserted and exposed to the headspace to extract the odour compounds. The samples were incubated at 60 °C for 40 min in the agitator at 1,500 rpm. Fibre desorption occurred after the fibre assembly was inserted into the injection port of the GC and held isothermally at 250 °C for 5 min.

The GC-MS analyses were performed on an Agilent 7890 gas chromatograph and a 5975C mass-selective detector. The GC separation employed a DB-5MS column of dimensions 60 m × 0.25 mm × 0.25 μm. The analysis was performed as previously described^69^. For SIM, the scan range, the m/z 40–400, and 5 ions were monitored: m/z 18 for water; m/z 95 and 108 for 2-methylisoborneol (MIB); and m/z 112 and 125 for geosmin (GSM). The retention times of 2-MIB and GSM, which were determined by an analysis of the calibration standards data, averaged 17.572 min and 23.843 min, respectively.

### Histopathology

Muscle samples were taken from the control and exposed fish. Small pieces (approximately 5 × 5 × 10 mm) were cut and placed in a fixative solution (Carnoy solution: 60% ethanol absolute, 30% chloroform, and 10% glacial acetic acid). After fixation (24–48 h), samples were dehydrated with ethanol absolute (2 h), ethanol:xylol (50:50% v/v) overnight and treated with xylol (4–5 h). Dehydrated samples were consecutively immersed in xylol/paraffin solutions of increasing paraffin concentration at 60 °C (1 h each) and in paraffin pure (2 h). Finally, samples were embedded in paraffin in small molds. Sections (4–5 mm thick) were obtained with a microtome (RM2145 Leica Microsystems, Bensheim, Germany) and fixed to glass plates with glycerin albumin in water (1/25 v/v), heating at 57 °C to melting the paraffin. After paraffin removing (xylol, twice, 10 min), solvent elimination (ethanol absolute) and rehydration (ethanol:water and pure water), sections were stained with hematoxylin and eosin (H&E)^70^. Stained sections were mounted with neutral balsam. Observations were carried out in a Zeiss microscope (Axioplan-2 imaging, USA).

### Extraction and determination of MCs

Muscle samples were taken from each group, and 5 g of muscle was separately homogenised in 100% methanol, stirred overnight at room temperature, and then centrifuged at 5000 rpm. The precipitates were reextracted twice with the same procedure, and the supernatants were combined. The detailed procedure was initially described by Zakaria^71^. The concentration of MCs concentration was analysed by the ELISA method, using microcystin plate kits (Envirologix Inc.®). Estimated daily intake (EDI) values of MCs in muscle were evaluated, and a coefficient of 5 was used to convert dry weight to wet weight according to Chen^54^. It is the EDI of MCs for an adult weighing 60 kg ingesting 300 g of edible organs of aquatic animals^54^. So the EDI = 1/5MCs (μg g^−1^DW)*300 g/60 kg.

### Statistics analysis

Every sample was measured in triplicates and average value was obtained from 3 fish in one tank. For each group, three values obtained from 3 tanks (including 9 fish) were used for statistical analysis. All results expressed as mean ± SD (n = 3) were subjected to one-way analysis of variance (ANOVA) and Duncan’s multiple comparison Test using STATISTICA software package (Version 6.0, Statsoft, Inc.). Differences were measured against control values and considered to be statistically significant at P < 0.05 level indicating with * and P < 0.01 level indicating **, respectively.

## Additional Information

**How to cite this article**: Liang, H. *et al.* Are fish fed with cyanobacteria safe, nutritious and delicious? A laboratory study. *Sci. Rep.* **5**, 15166; doi: 10.1038/srep15166 (2015).

## Figures and Tables

**Table 1:** Biometric parameters and proximate composition of goldfish (*Carassius auratus*) after 4, 8, 12 and 16 weeks of feeding with diets containing cyanobacteria lyophilized powder.

Time (weeks)	Diet	Weight (g)	Body length (cm)	Total length (cm)	Moisture (%)	Ash (%)	Crude protein (%)	Crude fat (%)
4	Control	84.15 ± 1.05	13.27 ± 0.07	16.66 ± 0.06	76.99 ± 0.31	7.10 ± 0.30	18.15 ± 0.11	4.06 ± 0.13
	10%	86.45 ± 1.05	13.45 ± 0.05	16.69 ± 0.09	77.15 ± 0.23	6.74 ± 0.13	18.26 ± 0.19	4.03 ± 0.03
	20%	89.60 ± 1.30*	13.69 ± 0.09**	16.86 ± 0.06	76.94 ± 0.08	6.65 ± 0.34	18.43 ± 0.09	3.80 ± 0.06
	30%	84.65 ± 1.05	13.66 ± 0.06*	16.86 ± 0.06	76.48 ± 0.67	7.04 ± 0.30	18.76 ± 0.05*	3.95 ± 0.06
	40%	87.35 ± 0.85	13.60 ± 0.10*	16.78 ± 0.08	77.48 ± 0.36	7.22 ± 0.15	18.44 ± 0.12	4.10 ± 0.07
8	Control	91.6 ± 0.80	14.26 ± 0.06	17.49 ± 0.09	75.14 ± 0.63	7.70 ± 0.23	18.30 ± 0.23	4.00 ± 0.10
	10%	95.55 ± 1.25	14.46 ± 0.06	17.96 ± 0.06**	75.06 ± 0.05	7.02 ± 0.27	18.31 ± 0.18	3.96 ± 0.08
	20%	102.45 ± 1.25**	14.65 ± 0.05*	18.08 ± 0.08**	75.86 ± 0.51	7.25 ± 0.25	18.58 ± 0.14	3.77 ± 0.08
	30%	90.75 ± 1.05	14.30 ± 0.10	17.56 ± 0.06	75.60 ± 0.46	6.55 ± 0.69	18.92 ± 0.05*	3.70 ± 0.01*
	40%	89.6 ± 1.10	14.09 ± 0.09	17.18 ± 0.08*	75.68 ± 0.21	6.85 ± 0.41	18.95 ± 0.05*	3.63 ± 0.06*
12	Control	104.25 ± 1.15	14.57 ± 0.07	17.78 ± 0.08	75.85 ± 0.38	7.62 ± 0.01	18.47 ± 0.10	3.99 ± 0.10
	10%	110.65 ± 0.85*	14.58 ± 0.08	17.86 ± 0.06	76.37 ± 0.07	7.37 ± 0.07	18.28 ± 0.12	3.84 ± 0.06
	20%	117.10 ± 1.80**	15.87 ± 0.07**	19.25 ± 0.05**	75.97 ± 0.44	7.61 ± 0.01	18.87 ± 0.09*	3.71 ± 0.05*
	30%	101.45 ± 0.75	14.86 ± 0.06*	17.76 ± 0.06	75.33 ± 0.59	7.51 ± 0.18	18.77 ± 0.05	3.66 ± 0.05*
	40%	94.7 ± 0.9**	14.48 ± 0.08	17.49 ± 0.09*	76.68 ± 0.24	7.09 ± 0.08*	18.74 ± 0.02	3.66 ± 0.05*
16	Control	109.15 ± 0.95	15.10 ± 0.10	18.35 ± 0.05	76.90 ± 0.62	8.89 ± 0.45	18.56 ± 0.16	3.89 ± 0.08
	10%	113.85 ± 0.95*	15.49 ± 0.09*	18.68 ± 0.08*	77.04 ± 0.11	8.97 ± 0.48	18.00 ± 0.11*	3.57 ± 0.10*
	20%	120.60 ± 0.80**	15.87 ± 0.07**	19.29 ± 0.09**	76.56 ± 0.35	8.16 ± 0.34	18.10 ± 0.18*	3.53 ± 0.08*
	30%	104.65 ± 1.05*	15.46 ± 0.06*	18.28 ± 0.08	77.52 ± 0.19	7.80 ± 0.12*	18.04 ± 0.07*	3.50 ± 0.08*
	40%	102.7 ± 1.10**	15.07 ± 0.07	17.78 ± 0.08**	76.97 ± 0.07	7.46 ± 0.08*	18.01 ± 0.01*	3.44 ± 0.06*

The values were expressed as mean ± SD (n = 3). Differences were measured against control values and considered to be statistically significant at P < 0.05 level indicating with * and P < 0.01 level indicating **, respectively.

**Table 2:** Fatty acid composition of goldfish (*Carassius auratus*) after 4, 8, 12 and 16 weeks of feeding with diets containing cyanobacteria lyophilized powder.

Time(weeks)	Diet	Fatty acids (% of total fatty acids)									
		C14:0	C15:0	C15:1	C16:0	C16:1	C17:0	C17:1	C18:0	C18:1	C18:2
4	Control	1.13 ± 0.07	2.00 ± 0.20	3.11 ± 0.47	26.85 ± 1.49	2.21 ± 0.13	2.25 ± 0.10	2.07 ± 0.14	13.54 ± 0.98	14.62 ± 1.25	6.94 ± 0.38
	10%	1.45 ± 0.13	1.92 ± 0.19	3.17 ± 0.45	27.90 ± 1.55	2.17 ± 0.10	2.25 ± 0.09	2.03 ± 0.16	13.76 ± 0.88	13.80 ± 1.45	7.02 ± 0.35
	20%	1.52 ± 0.16	1.79 ± 0.17	3.31 ± 0.47	29.15 ± 1.22	2.05 ± 0.09	2.15 ± 0.07	1.94 ± 0.10	14.33 ± 1.06	11.72 ± 0.35	7.18 ± 0.40
	30%	1.49 ± 0.05	1.69 ± 0.18	3.29 ± 0.46	29.74 ± 1.63	1.96 ± 0.10	2.13 ± 0.04	2.03 ± 0.11	15.31 ± 1.07*	11.24 ± 1.00	7.31 ± 0.43
	40%	1.34 ± 0.12	1.62 ± 0.17	3.89 ± 0.62	30.95 ± 1.42	1.84 ± 0.10	2.08 ± 0.04	1.95 ± 0.09	16.48 ± 1.21*	9.92 ± 0.64*	8.39 ± 0.40*
8	Control	1.12 ± 0.07	1.98 ± 0.16	3.13 ± 0.30	26.86 ± 0.52	2.21 ± 0.17	2.18 ± 0.16	2.00 ± 0.17	13.91 ± 0.64	14.40 ± 1.13	6.91 ± 0.30
	10%	1.36 ± 0.12	1.89 ± 0.04	3.29 ± 0.39	28.70 ± 1.42	2.05 ± 0.11	2.15 ± 0.07	2.08 ± 0.15	15.05 ± 0.72*	12.56 ± 1.29	7.11 ± 0.54
	20%	1.39 ± 0.12	1.70 ± 0.08	3.41 ± 0.48	29.69 ± 1.68	1.94 ± 0.08	2.18 ± 0.09	2.06 ± 0.18	16.08 ± 0.29*	12.67 ± 0.93	7.31 ± 0.44
	30%	1.38 ± 0.06	1.61 ± 0.04*	3.79 ± 0.25	31.85 ± 1.53*	1.89 ± 0.05	1.87 ± 0.06	1.96 ± 0.12	17.33 ± 0.93*	10.13 ± 0.75*	8.45 ± 0.34*
	40%	1.55 ± 0.12*	1.50 ± 0.06*	4.83 ± 0.45*	33.08 ± 1.30*	1.79 ± 0.04*	1.76 ± 0.08*	1.74 ± 0.10	17.55 ± 0.54**	9.56 ± 0.81*	8.50 ± 0.34*
12	Control	1.14 ± 0.07	1.92 ± 0.17	3.13 ± 0.39	27.24 ± 1.87	2.14 ± 0.10	2.22 ± 0.11	2.08 ± 0.06	13.46 ± 1.11	14.37 ± 1.00	7.03 ± 0.36
	10%	1.43 ± 0.09	1.81 ± 0.06	3.64 ± 0.27	30.19 ± 1.28	1.96 ± 0.05	2.01 ± 0.13	2.21 ± 0.05	17.87 ± 1.00*	10.72 ± 0.64	7.19 ± 0.53
	20%	1.46 ± 0.12*	1.66 ± 0.08	3.86 ± 0.21	33.76 ± 1.72*	1.90 ± 0.06*	1.73 ± 0.10*	2.30 ± 0.06	18.54 ± 1.45*	10.51 ± 1.86	7.35 ± 0.46
	30%	1.47 ± 0.04*	1.49 ± 0.06*	4.11 ± 0.26	34.94 ± 1.42*	1.82 ± 0.06*	1.68 ± 0.15*	2.23 ± 0.10	19.40 ± 0.82*	8.99 ± 0.61*	8.54 ± 0.34*
	40%	1.48 ± 0.04*	1.44 ± 0.06*	5.02 ± 0.44**	36.04 ± 1.31**	1.71 ± 0.03**	1.56 ± 0.07**	2.41 ± 0.06*	21.85 ± 1.42**	8.81 ± 1.43*	8.59 ± 0.36*
16	Control	1.14 ± 0.07	1.90 ± 0.20	3.11 ± 0.38	26.93 ± 0.45	2.18 ± 0.06	2.23 ± 0.11	2.01 ± 0.12	13.43 ± 0.81	14.41 ± 1.14	7.07 ± 0.60
	10%	1.52 ± 0.08*	1.53 ± 0.09	3.87 ± 0.22	31.57 ± 1.80	1.80 ± 0.04*	1.78 ± 0.16*	2.22 ± 0.21	18.30 ± 0.98*	9.85 ± 0.58*	7.27 ± 0.54
	20%	1.55 ± 0.13*	1.35 ± 0.14*	4.01 ± 0.28	35.06 ± 1.32*	1.74 ± 0.06*	1.54 ± 0.09**	2.34 ± 0.20	19.11 ± 1.03*	8.96 ± 0.67*	8.62 ± 0.32*
	30%	1.54 ± 0.11*	1.26 ± 0.08*	5.19 ± 0.61*	36.00 ± 1.38**	1.59 ± 0.09**	1.35 ± 0.08**	2.57 ± 0.13*	21.32 ± 0.93**	7.98 ± 1.39**	8.78 ± 0.25*
	40%	1.61 ± 0.04*	1.13 ± 0.08**	6.11 ± 0.44**	38.10 ± 2.28**	1.45 ± 0.14**	1.11 ± 0.13**	2.59 ± 0.04*	23.49 ± 1.88**	7.33 ± 1.06**	8.94 ± 0.28*
		**Fatty acids (% of total fatty acids)**									
		**C18:3**	**C20:4**	**C20:5**	**C22:6**	**SFA**	**MUFA**	**PUFA**	**n-3**	**n-6**	**n-3: n-6**
4	Control	2.48 ± 0.35	7.11 ± 0.58	3.87 ± 0.16	8.71 ± 0.22	45.77 ± 2.09	22.00 ± 0.79	29.11 ± 0.22	15.07 ± 0.74	14.05 ± 0.96	1.08 ± 0.13
	10%	2.43 ± 0.32	6.97 ± 0.46	3.88 ± 0.10	8.59 ± 0.14	47.26 ± 2.67	21.18 ± 1.06	28.89 ± 0.25	14.90 ± 0.56	13.99 ± 0.81	1.07 ± 0.10
	20%	2.34 ± 0.31	6.90 ± 0.73	3.79 ± 0.05	8.50 ± 0.03	48.95 ± 0.10	19.01 ± 1.01	28.71 ± 0.75	14.63 ± 0.39	14.07 ± 1.14	1.05 ± 0.11
	30%	1.90 ± 0.05	6.62 ± 0.50	3.60 ± 0.14	8.30 ± 0.02	50.36 ± 0.28	18.53 ± 1.66	27.73 ± 0.82	13.80 ± 0.11	13.93 ± 0.93	1.00 ± 0.07
	40%	1.79 ± 0.04	6.77 ± 0.30	3.42 ± 0.21	8.14 ± 0.05*	52.46 ± 0.03*	18.24 ± 1.93	28.52 ± 0.39	13.36 ± 0.31	15.16 ± 0.70	0.88 ± 0.06
8	Control	2.52 ± 0.25	7.09 ± 0.45	3.94 ± 0.08	8.70 ± 0.16	46.04 ± 0.50	21.74 ± 0.84	29.15 ± 0.25	15.16 ± 0.49	13.99 ± 0.75	1.09 ± 0.09
	10%	2.33 ± 0.32	6.78 ± 0.61	3.98 ± 0.06	8.57 ± 0.11	49.14 ± 1.91	19.99 ± 1.15	28.77 ± 0.66	14.87 ± 0.48	13.89 ± 1.15	1.08 ± 0.12
	20%	2.24 ± 0.28	6.64 ± 0.75	3.59 ± 0.04	8.31 ± 0.16	51.04 ± 1.84	20.09 ± 1.30	28.09 ± 0.71	14.14 ± 0.48	13.95 ± 1.19	1.02 ± 0.12
	30%	1.78 ± 0.08	6.42 ± 0.68	3.48 ± 0.15*	8.16 ± 0.06*	54.04 ± 2.50*	17.76 ± 1.18	28.29 ± 0.89	13.42 ± 0.13*	14.87 ± 1.02	0.91 ± 0.07
	40%	1.59 ± 0.08*	6.58 ± 0.24*	3.34 ± 0.20*	8.08 ± 0.05*	55.43 ± 0.61*	20.22 ± 1.00	28.08 ± 0.26	13.01 ± 0.17*	15.07 ± 0.10	0.86 ± 0.01
12	Control	2.52 ± 0.15	7.14 ± 0.50	3.83 ± 0.21	8.61 ± 0.24	45.98 ± 0.41	21.72 ± 0.56	29.12 ± 0.26	14.96 ± 0.60	14.16 ± 0.85	1.06 ± 0.11
	10%	2.24 ± 0.30	6.70 ± 0.14	3.60 ± 0.27	8.47 ± 0.10	53.29 ± 2.00	18.53 ± 0.27	28.21 ± 0.01	14.32 ± 0.68	13.89 ± 0.67	1.04 ± 0.10
	20%	2.16 ± 0.28	6.45 ± 0.10*	3.31 ± 0.03	8.08 ± 0.09*	57.14 ± 2.86*	18.57 ± 1.53	27.35 ± 0.16*	13.54 ± 0.40	13.81 ± 0.55	0.98 ± 0.07
	30%	1.64 ± 0.08*	6.13 ± 0.29*	3.21 ± 0.09*	8.11 ± 0.07*	58.98 ± 2.00**	17.15 ± 0.91	27.64 ± 0.69	12.97 ± 0.06*	14.67 ± 0.63	0.89 ± 0.03
	40%	1.43 ± 0.11*	5.75 ± 0.28	3.12 ± 0.11*	7.76 ± 0.08**	62.36 ± 2.55**	21.34 ± 3.56	26.65 ± 0.72*	12.31 ± 0.08**	14.34 ± 0.64	0.86 ± 0.03
16	Control	2.50 ± 0.26	7.17 ± 0.46	3.89 ± 0.14	8.75 ± 0.21	45.62 ± 0.73	21.72 ± 1.45	29.37 ± 0.44	15.13 ± 0.61	14.24 ± 1.05	1.07 ± 0.12
	10%	1.82 ± 0.16*	6.45 ± 0.18	3.32 ± 0.11*	8.40 ± 0.05	54.69 ± 2.45*	17.75 ± 1.05	27.27 ± 0.81	13.55 ± 0.10*	13.73 ± 0.71	0.99 ± 0.04
	20%	1.66 ± 0.07*	6.30 ± 0.11	3.25 ± 0.10**	7.90 ± 0.19**	58.60 ± 1.98*	17.05 ± 1.09	27.72 ± 0.43	12.81 ± 0.22**	14.91 ± 0.21	0.86 ± 0.01
	30%	1.50 ± 0.13**	6.00 ± 0.17	3.14 ± 0.10**	7.60 ± 0.06**	61.47 ± 2.05**	17.33 ± 0.56	27.03 ± 0.51*	12.25 ± 0.09**	14.79 ± 0.42	0.83 ± 0.02*
	40%	1.33 ± 0.13**	5.87 ± 0.18	2.96 ± 0.07**	7.57 ± 0.10**	65.44 ± 4.00**	17.74 ± 2.22	26.67 ± 0.63*	11.86 ± 0.16**	14.81 ± 0.46	0.80 ± 0.01*

The values were expressed as mean ± SD (n = 3). Differences were measured against control values and considered to be statistically significant at P < 0.05 level indicating with * and P < 0.01 level indicating **, respectively.

**Table 3:** Meat quality index of goldfish (*Carassius auratus*) after 4, 8, 12 and 16 weeks of feeding with diets containing cyanobacteria lyophilized powder.

Time (weeks)	Diet	muscle fibrediameter (μm)	myofibrilslength (μm)	cooking loss(%)	total collagen(μg/g)	pH
4	Control	46.38 ± 1.22	55.22 ± 1.15	22.39 ± 0.09	1896.97 ± 6.97	7.06 ± 0.07
	10%	46.66 ± 0.41	54.97 ± 1.02	22.17 ± 0.07	1910.01 ± 6.83	6.88 ± 0.09
	20%	47.79 ± 0.54	52.43 ± 2.43	22.49 ± 0.09	1892.65 ± 6.32	6.85 ± 0.08
	30%	47.54 ± 0.58	50.93 ± 1.73	22.79 ± 0.29	1906.30 ± 4.07	6.82 ± 0.09
	40%	48.29 ± 0.49	50.55 ± 1.48	22.95 ± 0.15	1865.97 ± 11.77*	6.80 ± 0.08
8	Control	47.96 ± 0.16	54.80 ± 0.73	23.10 ± 0.10	1896.07 ± 6.07	7.06 ± 0.08
	10%	46.94 ± 0.31	51.28 ± 1.38	23.07 ± 0.07	1908.78 ± 6.63	6.82 ± 0.10
	20%	48.24 ± 0.59	49.81 ± 1.31	23.16 ± 0.06	1879.37 ± 1.06	6.88 ± 0.03
	30%	48.66 ± 0.43	46.40 ± 3.55	23.07 ± 0.07	1896.35 ± 6.35	6.83 ± 0.05
	40%	50.11 ± 0.88*	42.63 ± 3.20*	23.80 ± 0.30*	1859.55 ± 10.25*	6.84 ± 0.04
12	Control	47.52 ± 1.50	53.09 ± 1.02	24.25 ± 0.05	1877.63 ± 7.63	7.00 ± 0.10
	10%	48.30 ± 1.01	49.02 ± 1.70	24.26 ± 0.06	1872.10 ± 2.80	6.84 ± 0.03
	20%	50.97 ± 0.09*	45.47 ± 0.87*	24.49 ± 0.06	1864.30 ± 5.40	6.86 ± 0.02
	30%	51.57 ± 0.36*	44.01 ± 3.05*	24.55 ± 0.13*	1825.25 ± 4.45*	6.80 ± 0.02
	40%	51.72 ± 0.34*	43.92 ± 1.72*	24.59 ± 0.02*	1847.80 ± 6.00*	6.79 ± 0.06*
16	Control	48.63 ± 2.30	54.36 ± 0.69	25.06 ± 0.06	1867.53 ± 7.53	6.99 ± 0.12
	10%	54.08 ± 0.28*	44.75 ± 0.70*	25.35 ± 0.05*	1846.75 ± 4.95*	6.80 ± 0.03
	20%	54.31 ± 0.71*	44.28 ± 0.40*	25.37 ± 0.05*	1843.91 ± 3.95*	6.76 ± 0.02*
	30%	54.78 ± 0.74*	42.11 ± 3.89*	25.58 ± 0.02**	1834.01 ± 4.45**	6.73 ± 0.01*
	40%	54.79 ± 0.46*	40.95 ± 3.88*	25.72 ± 0.08**	1824.32 ± 5.65**	6.69 ± 0.01*

The values were expressed as mean ± SD (n = 3). Differences were measured against control values and considered to be statistically significant at P < 0.05 level indicating with * and P < 0.01 level indicating **, respectively.

**Table 4:** Amino acid composition of goldfish (*Carassius auratus*) after 4, 8, 12 and 16 weeks of feeding with diets containing cyanobacteria lyophilized powder.

Time(weeks)	Diet	Amino acid (% of dry weight)									
		Asp	Thr	Ser	Glu	Gly	Ala	Cys	Val	Met	
4	Control	7.90 ± 0.10	3.52 ± 0.10	3.21 ± 0.16	12.35 ± 0.16	3.35 ± 0.06	4.45 ± 0.05	0.39 ± 0.01	3.78 ± 0.16	2.46 ± 0.10	
	10%	7.90 ± 0.02	3.67 ± 0.14	3.17 ± 0.11	12.32 ± 0.22	3.54 ± 0.06	4.70 ± 0.10	0.38 ± 0.03	3.82 ± 0.16	2.44 ± 0.08	
	20%	8.22 ± 0.21	3.83 ± 0.08	3.37 ± 0.11	12.91 ± 0.41	3.71 ± 0.09*	5.00 ± 0.11	0.35 ± 0.01	3.89 ± 0.14	2.42 ± 0.21	
	30%	8.36 ± 0.24	3.78 ± 0.14	3.49 ± 0.06	13.07 ± 0.47	3.82 ± 0.08*	5.14 ± 0.16*	0.38 ± 0.04	4.04 ± 0.18	2.59 ± 0.16	
	40%	8.16 ± 0.26	3.83 ± 0.05	3.38 ± 0.11	12.78 ± 0.58	3.45 ± 0.15	5.25 ± 0.35*	0.37 ± 0.01	4.14 ± 0.24	2.58 ± 0.04	
8	Control	8.01 ± 0.13	3.51 ± 0.13	3.27 ± 0.12	12.65 ± 0.05	3.70 ± 0.10	4.58 ± 0.18	0.39 ± 0.04	3.77 ± 0.08	2.39 ± 0.05	
	10%	8.24 ± 0.14	3.62 ± 0.10	3.35 ± 0.12	12.62 ± 0.32	3.72 ± 0.08	4.95 ± 0.07	0.35 ± 0.05	3.87 ± 0.15	2.50 ± 0.12	
	20%	8.33 ± 0.23	3.68 ± 0.07	3.38 ± 0.12	12.70 ± 0.60	3.75 ± 0.13	5.06 ± 0.14*	0.38 ± 0.06	4.03 ± 0.23	2.54 ± 0.19	
	30%	8.14 ± 0.09	3.50 ± 0.14	3.20 ± 0.12	12.42 ± 0.32	3.37 ± 0.17	4.88 ± 0.16	0.26 ± 0.02	4.13 ± 0.23	2.69 ± 0.17	
	40%	8.05 ± 0.17	3.40 ± 0.18	3.12 ± 0.12	11.18 ± 0.30*	3.27 ± 0.27	4.73 ± 0.07	0.25 ± 0.02*	4.32 ± 0.27	2.73 ± 0.11	
12	Control	8.25 ± 0.24	3.37 ± 0.15	3.28 ± 0.08	12.30 ± 0.29	3.49 ± 0.19	4.75 ± 0.15	0.38 ± 0.02	3.81 ± 0.06	2.34 ± 0.09	
	10%	8.15 ± 0.17	3.18 ± 0.04	3.08 ± 0.08	11.98 ± 0.05	3.33 ± 0.13	4.56 ± 0.05	0.35 ± 0.03	4.04 ± 0.22	2.55 ± 0.11	
	20%	8.11 ± 0.22	3.09 ± 0.03*	3.25 ± 0.10	11.94 ± 0.14	3.29 ± 0.29	4.86 ± 0.18	0.34 ± 0.06	4.21 ± 0.25	2.64 ± 0.13	
	30%	7.73 ± 0.12	3.17 ± 0.05	3.16 ± 0.08	10.60 ± 0.56*	2.60 ± 0.20*	3.83 ± 0.33	0.24 ± 0.01*	4.37 ± 0.25	2.82 ± 0.13*	
	40%	7.49 ± 0.06*	3.09 ± 0.02*	3.12 ± 0.03	10.46 ± 0.56*	3.25 ± 0.35	3.55 ± 0.54*	0.22 ± 0.01*	4.57 ± 0.21	2.85 ± 0.07*	
16	Control	7.85 ± 0.25	3.45 ± 0.19	3.16 ± 0.13	12.45 ± 0.15	3.63 ± 0.08	4.43 ± 0.18	0.39 ± 0.02	3.81 ± 0.11	2.37 ± 0.09	
	10%	7.78 ± 0.18	3.13 ± 0.07	2,90 ± 0.10	11.50 ± 0.50	3.61 ± 0.10	4.15 ± 0.12	0.38 ± 0.02	4.16 ± 0.22	2.63 ± 0.11	
	20%	7.58 ± 0.08	3.04 ± 0.06*	2.84 ± 0.10	10.78 ± 0.18	3.17 ± 0.37	3.99 ± 0.19	0.30 ± 0.03*	4.28 ± 0.25	2.74 ± 0.09*	
	30%	7.26 ± 0.13*	2.95 ± 0.11*	2.48 ± 0.25*	9.90 ± 0.90*	3.46 ± 0.26	3.40 ± 0.30*	0.24 ± 0.02**	4.54 ± 0.29	2.84 ± 0.13*	
	40%	7.16 ± 0.09*	2.72 ± 0.06**	2.38 ± 0.20*	9.75 ± 0.85*	2.65 ± 0.15*	3.31 ± 0.30*	0.20 ± 0.02**	4.68 ± 0.25*	2.95 ± 0.10**	
		**Amino acid (% of dry weight)**									
		Ile	Leu	Tyr	Phe	Lys	His	Arg	Pro	Flavor aa	Total aa
4	Control	3.42 ± 0.04	6.33 ± 0.07	2.39 ± 0.14	4.22 ± 0.14	7.03 ± 0.10	2.60 ± 0.11	4.34 ± 0.14	2.15 ± 0.11	28.04 ± 0.36	73.84 ± 0.74
	10%	3.40 ± 0.05	6.23 ± 0.29	2.56 ± 0.09	4.00 ± 0.12	6.90 ± 0.10	2.53 ± 0.11	4.47 ± 0.17	2.12 ± 0.12	28.46 ± 0.08	74.12 ± 0.23
	20%	2.97 ± 0.46	6.03 ± 0.19	2.67 ± 0.10	4.15 ± 0.17*	6.82 ± 0.10	2.51 ± 0.09	4.72 ± 0.12	2.24 ± 0.12	29.83 ± 0.42*	75.78 ± 0.58*
	30%	3.48 ± 0.04	6.29 ± 0.20	2.76 ± 0.10	4.07 ± 0.13*	6.74 ± 0.11	2.58 ± 0.22	4.84 ± 0.14*	2.41 ± 0.08	30.39 ± 0.47*	77.82 ± 0.08*
	40%	3.49 ± 0.13	6.20 ± 0.18	2.64 ± 0.13	4.01 ± 0.19*	6.66 ± 0.12	2.46 ± 0.14	4.86 ± 0.06*	2.54 ± 0.05*	29.64 ± 0.64*	76.78 ± 0.39*
8	Control	3.39 ± 0.03	6.36 ± 0.21	2.40 ± 0.10	4.23 ± 0.09	7.13 ± 0.12	2.58 ± 0.07	4.40 ± 0.19	2.17 ± 0.07	28.94 ± 0.26	74.90 ± 1.04
	10%	3.44 ± 0.06	6.23 ± 0.08	2.50 ± 0.02	4.35 ± 0.07	6.83 ± 0.08	2.71 ± 0.05	4.57 ± 0.27	2.38 ± 0.08	29.53 ± 0.31	76.21 ± 0.65
	20%	3.33 ± 0.11	6.33 ± 0.11	2.65 ± 0.06*	4.51 ± 0.12	6.81 ± 0.11	2.71 ± 0.09	4.73 ± 0.13	2.60 ± 0.05*	29.84 ± 0.56	77.49 ± 0.68
	30%	3.42 ± 0.04	6.20 ± 0.15	2.58 ± 0.06	4.40 ± 0.14	6.75 ± 0.09*	2.69 ± 0.06	4.51 ± 0.21	1.97 ± 0.12	28.81 ± 0.42	75.07 ± 0.81
	40%	3.46 ± 0.10	6.15 ± 0.17	2.70 ± 0.05*	4.62 ± 0.12	6.66 ± 0.09*	2.79 ± 0.11	4.31 ± 0.31	1.80 ± 0.04*	27.23 ± 0.67*	73.49 ± 0.97
12	Control	3.31 ± 0.03	6.41 ± 0.18	2.45 ± 0.06	4.16 ± 0.13	7.22 ± 0.09	2.59 ± 0.13	4.39 ± 0.19	2.20 ± 0.04	28.78 ± 0.86	74.66 ± 1.83
	10%	3.40 ± 0.25	6.70 ± 0.05	2.52 ± 0.07	3.74 ± 0.08	6.75 ± 0.16	2.72 ± 0.10	4.19 ± 0.19	2.06 ± 0.06	28.01 ± 0.13	73.28 ± 0.24
	20%	3.56 ± 0.16	6.79 ± 0.06	2.72 ± 0.10	4.04 ± 0.10*	6.54 ± 0.19*	2.79 ± 0.11	3.53 ± 0.23*	1.88 ± 0.09*	28.19 ± 0.04	73.55 ± 0.52
	30%	3.61 ± 0.17*	6.80 ± 0.08	2.80 ± 0.09*	4.48 ± 0.16	6.48 ± 0.20*	2.73 ± 0.12	3.43 ± 0.23*	1.82 ± 0.09*	24.75 ± 1.20*	70.64 ± 1.58
	40%	3.56 ± 0.21	6.49 ± 0.16	2.88 ± 0.06*	4.62 ± 0.10	6.35 ± 0.21*	2.86 ± 0.10	3.37 ± 0.21*	1.79 ± 0.10*	24.74 ± 1.50*	70.48 ± 1.78
16	Control	3.34 ± 0.12	6.33 ± 0.11	2.47 ± 0.06	4.15 ± 0.06	7.05 ± 0.07	2.60 ± 0.10	4.24 ± 0.16	2.11 ± 0.13	28.35 ± 0.35	73.81 ± 1.26
	10%	3.47 ± 0.06	6.45 ± 0.07	2.55 ± 0.07	4.37 ± 0.04	6.57 ± 0.13*	2.74 ± 0.09	3.57 ± 0.14	1.82 ± 0.08	27.03 ± 0.30	71.74 ± 0.16
	20%	3.64 ± 0.10	6.72 ± 0.09*	2.78 ± 0.10	4.61 ± 0.16	6.51 ± 0.10*	2.84 ± 0.10	3.44 ± 0.24*	1.72 ± 0.09*	25.52 ± 0.82	70.96 ± 1.23
	30%	3.75 ± 0.07*	6.81 ± 0.09*	2.84 ± 0.14*	4.77 ± 0.15	6.37 ± 0.15**	2.80 ± 0.08	3.34 ± 0.24*	1.63 ± 0.10*	24.02 ± 1.59*	69.35 ± 1.53
	40%	3.82 ± 0.11*	6.72 ± 0.10*	2.92 ± 0.07*	4.69 ± 0.13*	6.28 ± 0.08**	2.95 ± 0.07*	3.20 ± 0.15*	1.55 ± 0.09*	22.86 ± 1.38*	67.87 ± 1.83*

The values were expressed as mean ± SD (n = 3). Differences were measured against control values and considered to be statistically significant at P < 0.05 level indicating with * and P < 0.01 level indicating **, respectively.

**Table 5:** The organic acids composition of goldfish (*Carassius auratus*) after 4, 8, 12 and 16 weeks of feeding with diets containing cyanobacteria lyophilized powder.

Time(weeks)	Diet	Organic acid (μg/g)					
		Propionic acid	Pyruvic acid	Oxalic acid	Succinic acid	Lactic acid	Acetic acid
4	Control	96.30 ± 3.83	33.94 ± 1.29	125.10 ± 7.90	147.36 ± 4.90	192.52 ± 11.72	64.08 ± 0.85
	10%	95.79 ± 4.26	33.89 ± 0.76	120.12 ± 4.29	146.06 ± 5.48	194.46 ± 12.27	64.47 ± 1.19
	20%	107.04 ± 4.21	38.61 ± 1.87	144.95 ± 11.50	147.51 ± 3.24	185.55 ± 7.19	66.08 ± 0.71
	30%	121.13 ± 4.94*	39.34 ± 1.98	156.32 ± 12.50	149.79 ± 3.27	207.07 ± 6.15	68.48 ± 1.21*
	40%	122.72 ± 9.99*	38.53 ± 1.75	148.29 ± 11.65	162.18 ± 5.64	197.74 ± 5.00	69.32 ± 1.05*
8	Control	99.51 ± 9.52	33.78 ± 0.49	124.57 ± 6.34	148.74 ± 16.37	199.01 ± 10.02	63.92 ± 1.19
	10%	102.83 ± 9.19	32.93 ± 0.69	130.27 ± 6.52	168.37 ± 11.82	200.85 ± 11.38	71.68 ± 0.69**
	20%	121.56 ± 8.99	38.89 ± 1.43*	181.89 ± 9.16*	155.36 ± 14.72	191.27 ± 11.36	71.97 ± 1.57**
	30%	96.43 ± 11.88	33.00 ± 0.53	171.10 ± 7.35*	114.47 ± 6.82	212.48 ± 9.90	72.91 ± 0.54**
	40%	139.66 ± 3.98*	39.59 ± 2.34*	184.17 ± 20.42*	153.21 ± 16.66	233.41 ± 12.33	70.90 ± 1.46**
12	Control	100.97 ± 1.41	33.32 ± 1.94	125.57 ± 6.43	142.98 ± 19.75	201.83 ± 9.09	64.28 ± 1.00
	10%	123.13 ± 10.40	37.72 ± 1.47	150.14 ± 3.50	146.17 ± 19.63	207.11 ± 14.27	68.36 ± 1.08*
	20%	139.72 ± 7.08*	37.74 ± 1.19	188.46 ± 19.58*	154.78 ± 21.76	231.06 ± 14.77	74.23 ± 1.04**
	30%	145.81 ± 7.97*	34.28 ± 1.90	195.87 ± 15.74*	138.93 ± 17.61	207.77 ± 6.08	69.47 ± 0.80*
	40%	180.35 ± 12.49**	37.94 ± 1.69	188.32 ± 17.10*	131.43 ± 14.90	252.99 ± 10.76*	77.01 ± 1.37**
16	Control	96.49 ± 3.75	33.55 ± 1.82	124.83 ± 6.67	148.45 ± 4.28	196.13 ± 6.11	63.75 ± 0.53
	10%	95.95 ± 4.09	38.36 ± 1.08	159.21 ± 3.14	145.95 ± 6.40	220.28 ± 15.26	68.70 ± 1.59*
	20%	130.86 ± 1.37*	33.08 ± 1.46	172.38 ± 14.98*	133.50 ± 9.03	232.35 ± 7.89	75.47 ± 0.90**
	30%	132.71 ± 11.03*	47.93 ± 2.57**	125.12 ± 12.28	156.27 ± 7.18	247.75 ± 14.62*	70.37 ± 0.87**
	40%	139.67 ± 6.92**	47.36 ± 1.09**	134.26 ± 11.52	165.39 ± 10.05	263.19 ± 16.84*	71.59 ± 0.79**

The values were expressed as mean ± SD (n = 3). Differences were measured against control values and considered to be statistically significant at P < 0.05 level indicating with * and P < 0.01 level indicating **, respectively.

**Table 6:** The nucleotide compounds composition of goldfish (*Carassius auratus*) after 4, 8, 12 and 16 weeks of feeding with diets containing cyanobacteria lyophilized powder.

Time(weeks)	Diet	Nucleotides (μg/g)								
		ATP	ADP	AMP	CMP	GMP	IMP	UMP	Hx	HxR
4	Control	15.62 ± 0.38	57.85 ± 2.48	54.04 ± 1.73	35.16 ± 2.80	1192.24 ± 21.51	1539.63 ± 36.79	52.96 ± 1.31	61.04 ± 3.68	490.44 ± 14.32
	10%	15.52 ± 0.35	56.06 ± 2.43	53.35 ± 2.54	34.81 ± 2.93	1191.86 ± 10.87	1550.49 ± 37.86	53.79 ± 1.58	62.20 ± 4.35	495.69 ± 18.08
	20%	15.45 ± 0.28	57.05 ± 2.80	56.03 ± 1.66	33.27 ± 2.44	1182.91 ± 9.93	1534.90 ± 42.16	53.12 ± 1.08	63.21 ± 4.16	502.95 ± 19.69
	30%	15.34 ± 0.30	55.18 ± 2.80	54.50 ± 1.14	30.96 ± 1.60	1163.00 ± 9.73	1513.94 ± 31.21	51.71 ± 0.45	64.14 ± 3.76	511.78 ± 20.96
	40%	14.43 ± 0.30*	55.30 ± 1.29	53.52 ± 1.89	29.83 ± 2.00	1153.54 ± 9.20	1511.16 ± 11.57	50.72 ± 0.44	65.61 ± 4.06	520.23 ± 11.49
8	Control	15.58 ± 0.26	56.00 ± 2.27	52.77 ± 1.51	34.90 ± 1.63	1193.74 ± 30.00	1537.69 ± 24.95	53.04 ± 2.39	59.77 ± 3.50	487.64 ± 25.00
	10%	15.40 ± 0.22	55.30 ± 2.66	53.86 ± 1.49	32.46 ± 1.93	1184.26 ± 18.38	1544.28 ± 30.64	51.77 ± 2.22	63.16 ± 4.22	501.60 ± 22.19
	20%	15.70 ± 0.23	54.44 ± 2.82	52.13 ± 1.84	30.25 ± 1.87	1166.26 ± 16.48	1530.60 ± 37.86	50.79 ± 2.03	64.66 ± 3.78	519.75 ± 12.99
	30%	15.64 ± 0.40*	54.29 ± 3.05	50.56 ± 1.17	29.00 ± 2.62	1149.85 ± 12.89	1508.19 ± 45.83	49.47 ± 2.20	71.01 ± 1.38*	530.65 ± 13.10
	40%	15.69 ± 0.32*	54.23 ± 1.50	46.79 ± 1.32*	28.10 ± 2.74	1131.04 ± 11.60	1415.67 ± 12.79*	48.43 ± 2.16	72.65 ± 1.10*	561.47 ± 21.27*
12	Control	15.61 ± 0.33	56.96 ± 2.42	52.81 ± 1.57	35.20 ± 2.05	1190.01 ± 18.79	1540.75 ± 31.99	52.84 ± 0.83	60.55 ± 3.71	482.79 ± 20.05
	10%	15.88 ± 0.24	55.38 ± 2.84	47.37 ± 3.54	31.01 ± 1.17	1161.42 ± 11.42	1483.02 ± 30.28	50.93 ± 0.19	68.79 ± 1.74*	520.01 ± 22.63
	20%	14.70 ± 0.10*	52.44 ± 2.93	47.81 ± 0.57	29.51 ± 2.13	1148.99 ± 23.85	1488.33 ± 35.59	50.82 ± 0.80	70.55 ± 1.19*	532.67 ± 31.08
	30%	14.54 ± 0.23*	45.65 ± 3.43*	45.43 ± 1.06*	28.57 ± 2.03*	1123.52 ± 9.22*	1432.29 ± 17.56*	49.50 ± 1.23	71.59 ± 0.68*	594.74 ± 29.01*
	40%	14.32 ± 0.25*	44.11 ± 3.03*	44.49 ± 0.97*	26.72 ± 1.36*	1114.21 ± 9.54*	1391.77 ± 10.97*	47.56 ± 1.29*	76.06 ± 1.10**	616.91 ± 36.84*
16	Control	15.63 ± 0.24	57.82 ± 2.46	53.81 ± 1.43	34.54 ± 1.70	1193.19 ± 30.45	1541.37 ± 27.59	52.85 ± 1.71	59.57 ± 3.68	482.79 ± 30.05
	10%	14.73 ± 0.19*	48.11 ± 1.98*	47.15 ± 1.12*	29.79 ± 1.41	1153.68 ± 29.16	1466.13 ± 18.45*	50.20 ± 0.34	69.98 ± 1.75*	527.30 ± 25.97
	20%	14.61 ± 0.18*	45.79 ± 3.42*	46.12 ± 0.96*	27.42 ± 1.06*	1110.57 ± 13.16*	1437.93 ± 25.10*	48.80 ± 1.54	71.56 ± 1.08**	590.19 ± 22.64*
	30%	14.38 ± 0.30*	42.56 ± 2.28**	42.17 ± 1.81**	25.58 ± 1.22**	1097.96 ± 15.92*	1416.77 ± 14.03**	47.81 ± 1.08*	73.56 ± 0.59**	614.01 ± 30.16*
	40%	14.13 ± 0.30**	40.61 ± 1.48**	40.05 ± 1.58**	23.14 ± 1.86**	1075.60 ± 12.87*	1375.80 ± 7.04**	46.52 ± 0.89*	77.02 ± 1.31**	652.10 ± 30.74**

The values were expressed as mean ± SD (n = 3). Differences were measured against control values and considered to be statistically significant at P < 0.05 level indicating with * and P < 0.01 level indicating **, respectively.

**Table 7:** The carnosine levels of goldfish (*Carassius auratus*) after 4, 8, 12 and 16 weeks of feeding with diets containing cyanobacteria lyophilized powder.

Time (weeks)	Carnosine (mg/g)				
	Control	10%	20%	30%	40%
4	13.36 ± 0.71	13.52 ± 0.95	14.25 ± 0.81	15.89 ± 0.22*	16.79 ± 0.26*
8	13.16 ± 0.50	13.56 ± 0.29	15.63 ± 0.90	16.40 ± 1.25*	17.36 ± 0.69*
12	13.26 ± 0.41	13.96 ± 0.29	17.65 ± 0.88*	18.94 ± 1.14*	20.50 ± 2.07**
16	13.35 ± 0.30	14.64 ± 0.53	19.56 ± 1.49*	22.48 ± 1.98**	25.92 ± 1.42**

The values were expressed as mean ± SD (n = 3). Differences were measured against control values and considered to be statistically significant at P < 0.05 level indicating with * and P < 0.01 level indicating **, respectively.

**Table 8:** Composition of diets in control and cyanobacteria groups.

Ingredient (g)/100 g of feed	Control	10%	20%	30%	40%
Soybean meal (oil-extracted)	70	60	50	40	30
Fish meal	15	15	15	15	15
Fish oil	0	0.5	1.1	1.6	2.1
Corn starch	6	5.72	5.33	5.07	4.8
Carboxymethyl cellulose	3.89	3.67	3.46	3.22	2.99
Vitamin-mineral mix	5	5	5	5	5
Vitamin C	0.01	0.01	0.01	0.01	0.01
Choline chloride	0.1	0.1	0.1	0.1	0.1
Cyanobacteria lyophilized powder	0	10	20	30	40
